# Supplementary material for: A Disposable Electrochemical Biosensor Based on Screen-Printed Carbon Electrodes Modified with Silver Nanowires/HPMC/Chitosan/Urease for the Detection of Mercury (II) in Water
Source: Biosensors (Basel). 2021 Sep 23;11(10):351. doi: 10.3390/bios11100351 (PMC8534075; doi:10.3390/bios11100351)
Supplement: Supplementary file 1 [file biosensors-11-00351-s001.zip › biosensors-1362372-supplementary.pdf]

biosensors

Supplementary Materials

A disposable electrochemical biosensor based onscreen-printed carbon electrodes modified with silver nanowires/HPMC/chitosan/urease for the detection of mercury(II) in water

Apichart Saenchoopa[1], Supannika Klangphukhiew[2], Rachata Somsub[3], Chanon Talodthaisong[1], Rina Patramanon[2], Jureerut Daduang[4], Sakda Daduang[5,*], Sirinan Kulchat[1,*]

1 Department of Chemistry, Faculty of Science, Khon Kaen University, Khon Kaen 40002, Thailand; apichats@kkumail.com (A.S.); chanon@kkumail.com (C.T.)
2 Department of Biochemistry, Faculty of Science, Khon Kaen University, Khon Kaen 40002, Thailand; supannika.k@kkumail.com (S.K.); narin@kku.ac.th (R.P.)
3 Department of Physics, Faculty of Science, Khon Kaen University, Khon Kaen 40002, Thailand; petch.rachataa@gmail.com
4 Department of Clinical Chemistry, Faculty of Associated Medical Sciences, Khon Kaen University, KhonKaen 40002, Thailand; jurpoo@kku.ac.th
5 Division of Pharmacognosy and Toxicology, Faculty of Pharmaceutical Sciences, Khon Kaen University, Khon Kaen 40002, Thailand
* Correspondence: sakdad@kku.ac.th (S.D.); sirikul@kku.ac.th (S.K.)

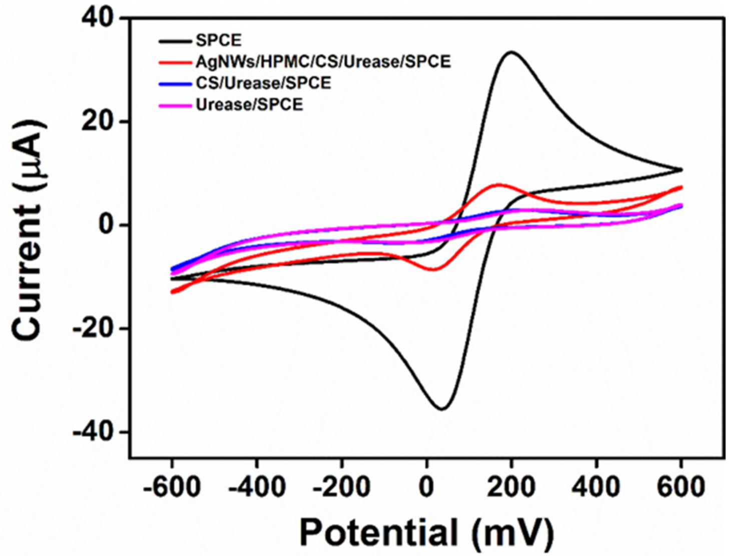

Figure S1. CV response of bare SPCE (black), Urease/SPCE (pink), CS/Urease/SPCE (blue), and AgNWs/HPMC/CS/Urease/SPCE (red) in the presence of 5 mM $K_3[Fe(CN)_6]$/0.1 M KCl.

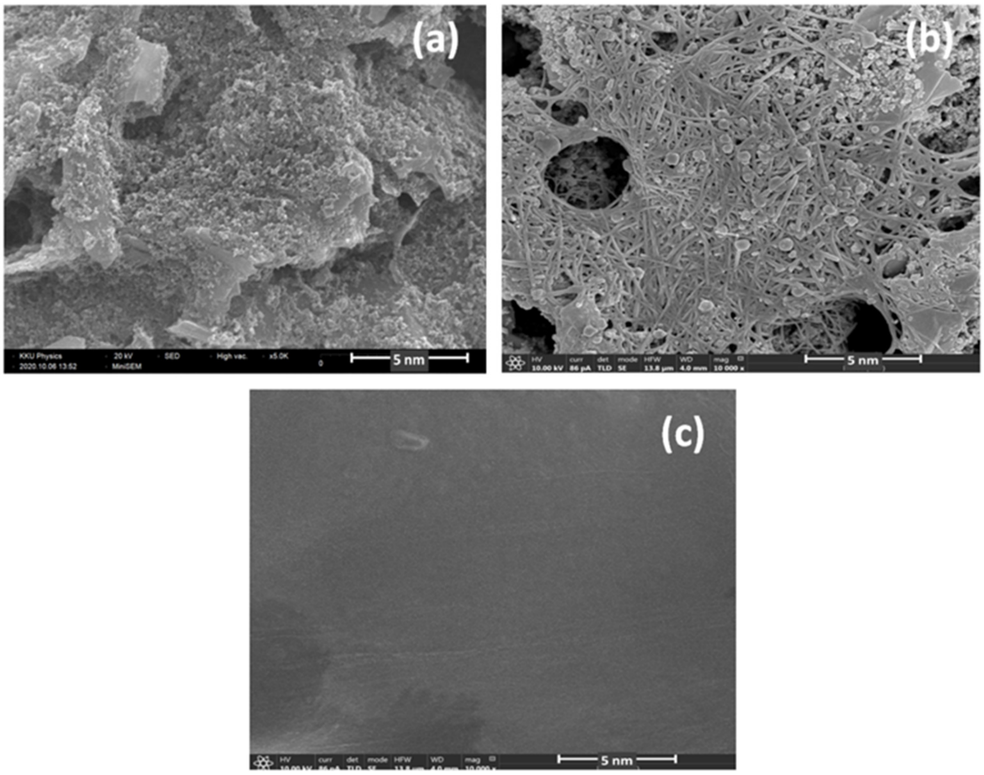

Figure S2. SEM images of **(a)** bare SPCE, **(b)** AgNWs modified SPCE, and **(c)** AgNWs/HPMC/CS/ Urease modified SPCE.

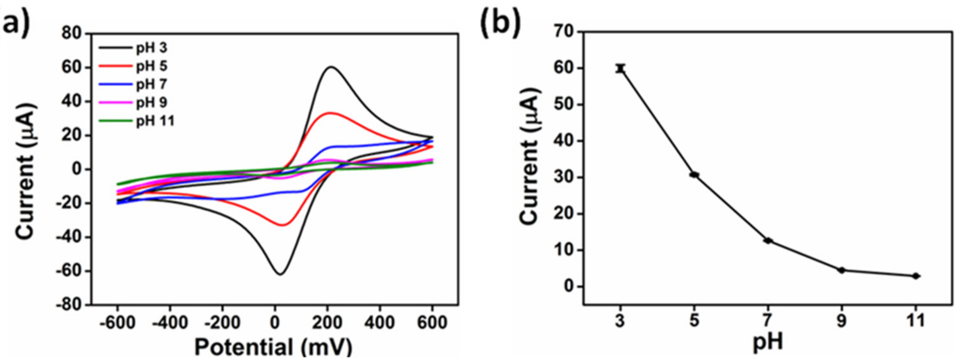

Figure S3. (a) CV response of AgNWs/CS/Urease at different pH ranging from 3, 5, 7, 9 and 11 in the presence of 5 mM $K_3[Fe(CN)_6]$/0.1 M KCl, 20 μM Hg^{2+} and scan rate 100 mVs^{-1}. **(b)** The anodic current (I_{pa}) as a function of Ph

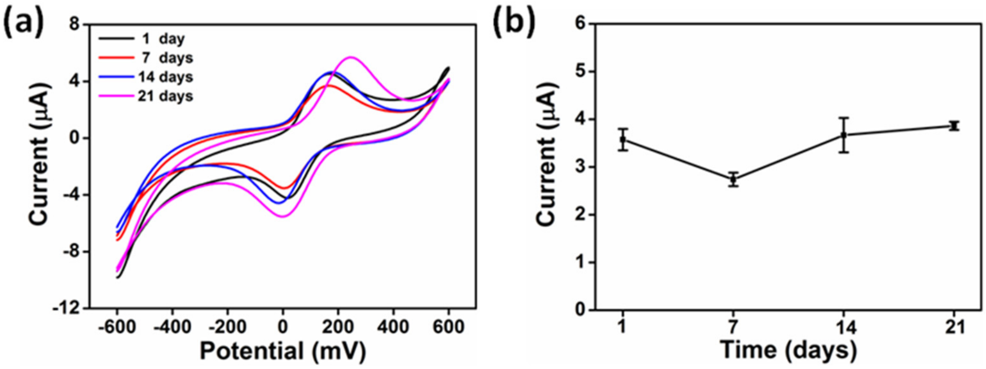

Figure S4. (a) CV response of AgNWs/CS/Urease at different days ranging from 1, 7, 14 and 21 days in the presence of 5 mM $K_3[Fe(CN)_6]$/0.1 M KCl, 20 μM Hg^{2+} and scan rate 100 mVs^{-1}. **(b)** The anodic current (I_{pa}) as a functions of day.

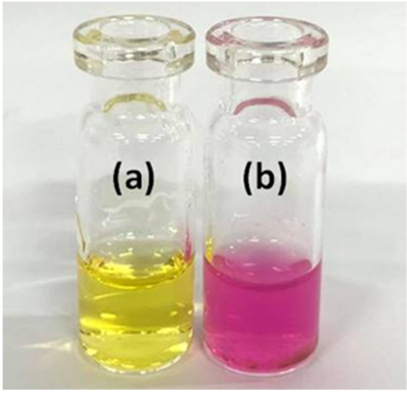

Figure S5. Activity test of Urease **(a)** 0.05 mM phenol red 1.0 mL (blank solution, yellow), **(b)** 0.05 mM phenol red in the presence of 2.5 μg/μL urease and 2 %w/v urea after incubation for 10 min, the color changed from yellow to pink indicating that ammonium species were produced during the enzymatic reaction process [1].

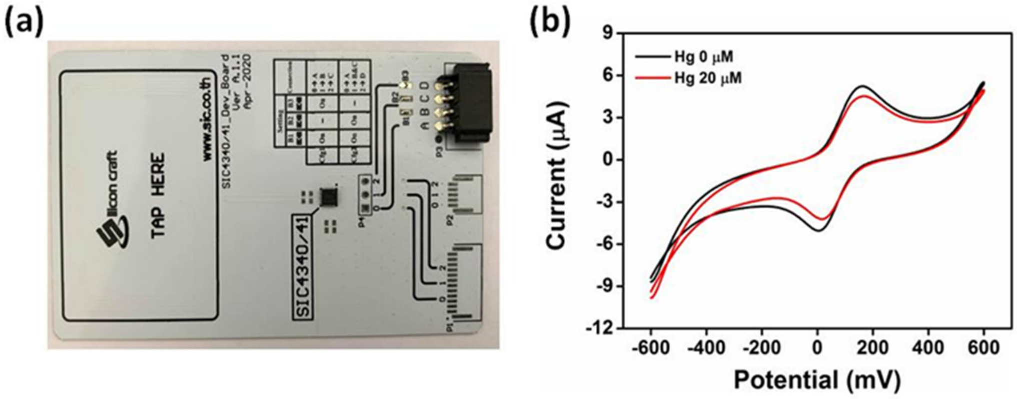

Figure S6. (a) A near-field communication (NFC) potentiostat planar antenna (NFC microchip SIC4340/41), **(b)** Cyclic votammogram (CV) response of modified electrode in the presence of 20 μM Hg^{2+} (red) and in the absence of Hg^{2+} (black) in 2 %w/v urea, and 5 mM $K_3[Fe(CN)_6]$/0.1 M KCl sweeping the potential between –600 mV to 600 mV, at a scan rate of 100 mV/s using the NFC potentiostat as reported previously [2]. The current was reduced in the presence of 20 μM Hg^{2+}. The results showed good agreement of cyclic voltammogram with those using the electrochemical workstation ECAS100.

Reference:

1. Tavares, M.C.; Oliveira, K.A.; de Fátima, Â.; Coltro, W.K.T.; Santos, J.C.C. Paper-based analytical device with colorimetric detection for urease activity determination in soils and evaluation of potential inhibitors. *Talanta*. **2021**, *230*, 122301. doi: 10.1016/j.talanta.2021.122301.
2. Krorakai, K.; Klangphukhiew, S.; Kulchat, S.; Patramanon, R. Smartphone-based NFC potentiostat for wireless electrochemical sensing. *Appl. Sci.* **2021**, *11*. doi: 10.3390/app11010392.
